# Dyslipidemia, Insulin Resistance, Ectopic Lipid Accumulation, and Vascular Function in Resistance to Thyroid Hormone β

**DOI:** 10.1210/clinem/dgab002

**Published:** 2021-02-01

**Authors:** Carla Moran, Carmel M McEniery, Nadia Schoenmakers, Catherine Mitchell, Alison Sleigh, Laura Watson, Greta Lyons, Keith Burling, Peter Barker, Krishna Chatterjee

**Affiliations:** 1 Wellcome Trust-MRC Institute of Metabolic Science, University of Cambridge, Cambridge, UK; 2 Division of Experimental Medicine and Immunotherapeutics, University of Cambridge, Cambridge, UK; 3 Department of Endocrinology, Hillingdon Hospital, Hillingdon, UK; 4 Wolfson Brain Imaging Centre, University of Cambridge School of Clinical Medicine, Cambridge, UK; 5 NIHR Cambridge BRC Core Biochemical Assay Laboratory, Cambridge University Hospitals NHS Foundation Trust, Cambridge, UK

**Keywords:** dyslipidemia, hepatic steatosis, resistance to thyroid hormone beta, vascular function, thyroid hormone receptor

## Abstract

**Purpose:**

In resistance to thyroid hormone due to mutations in thyroid hormone receptor β, peripheral tissues are variably refractory to the action of circulating thyroid hormones. We evaluated parameters contributing to atherosclerotic risk in this disorder.

**Methods:**

We measured low-density lipoprotein cholesterol (LDL-C), triglyceride (TG), high-density lipoprotein cholesterol (HDL-C), nonesterified fatty acids (NEFA), intrahepatic lipid (IHL) and intramyocellular lipid (IMCL), Homeostasis-model assessment of insulin resistance (HOMA-IR), augmentation index (AIx) and pulse wave velocity (PWV), flow-mediated dilatation, and carotid intima-media thickness (cIMT) in an unselected, genetically confirmed cohort of adult RTHβ patients (n = 27-77) and compared these with measurements in healthy subjects (up to n = 100) and thyrotoxic patients (n = 40).

**Results:**

Resistance to thyroid hormone beta (RTHβ) patients exhibited higher LDL-C (*P* = 0.008) and TG (*P* = 0.002) and lower HDL-C concentrations (*P* = 0.015 × 10^–2^) than control subjects, with LDL-C being higher than in thyrotoxic patients with comparable hyperthyroxinemia. Proprotein convertase subtilisin/kexin 9 (*P* = 0.002) and apolipoprotein B (*P* = 0.0009) levels were reduced in thyrotoxic patients but not lower in RTHβ patients or control subjects. Intrahepatic lipid (*P* = 0.02 × 10^–4^), IMCL (*P* = 0.002), HOMA-IR (*P* = 0.01 × 10^–2^), and NEFA (*P* = 0.04 × 10^–6^) were significantly higher in RTHβ patients than control subjects. Flow-mediated dilatation was increased (*P* = 0.04) but cIMT (*P* = 0.71), PWV *P* = 0.81), and AIx (*P* = 0.95) were unaltered in RTHβ patients.

**Conclusions:**

We have documented mixed dyslipidemia with hepatic and IMCL accumulation in RTHβ, suggesting that surveillance for these metabolic abnormalities is warranted. How they combine with enhanced endothelial function and unaltered vessel wall thickness and compliance to determine overall cardiometabolic risk in this disorder remains to be defined.

Thyroid hormones (TH) regulate physiological processes via separate receptor genes (*THRA, THRB*) that are alternately spliced to generate receptor subtypes (TRα1; TRβ2, TRβ1) which are most highly expressed in different tissues (TRβ2: hypothalamus and pituitary; TRβ1, liver; TRα1, central nervous system, myocardium, skeletal muscle, gastrointestinal tract, white adipose tissue) (1). With an estimated prevalence of approximately 1 in 40 000, about 3000 individuals from approximately 1000 families with the genetic disorder of resistance to thyroid hormone β (RTHβ) have been identified worldwide (2). In this condition, heterozygous, mutant TRβ inhibits action of wild type receptor in a dominant negative manner (3); such inhibition of TRβ action within the hypothalamic-pituitary thyroid axis results in elevated circulating TH with nonsuppressed TSH levels, characteristic of the disorder (4).

Many clinical and biochemical characteristics of RTHβ are known to be governed by the thyroid status of different peripheral tissues, which, in turn, is significantly mediated by the predominant receptor subtype (TRα *vs* TRβ) expressed in that target organ. Thus, raised metabolic rate in RTHβ reflects hyperresponsiveness of TRα1-expressing skeletal muscle to elevated circulating TH (5). Likewise, increased heart rate and cardiac contractility seen in adults and children with RTHβ, together with atrial fibrillation in older patients, suggests a relatively hyperthyroid state in TRα1-expressing myocardium (6). Conversely, in contrast to elevated levels in thyrotoxicosis (TT), circulating concentrations of sex-hormone binding globulin (a hepatic marker of TH action) and coagulation pathway proteins are normal in RTHβ (7,8), suggesting resistance to hormone action and a relative hypothyroid state in the liver in this disorder.

Previous studies have correlated clinical phenotypes (eg, circulating free T4 levels, resting energy expenditure) in RTHβ with *THRB* mutation genotype, but only in subjects with TRβ mutations whose transcriptional properties are proportional to the magnitude of impaired T3 binding (so-called type 1 mutations), whereas individuals with TRβ mutations whose transcriptional activity is not related to T3 binding (so-called type 2 mutations) show no such correlation (4,5).

Conventional hypothyroidism is associated with raised serum total and low-density lipoprotein cholesterol (LDL-C) concentrations and also raised triglyceride levels (9,10). Both overt and subclinical hypothyroidism have been associated with an increased occurrence of nonalcoholic fatty liver disease (11,12). Hypothyroidism is also associated with reduced arterial vascular compliance, impaired endothelial function and increased carotid artery intima media thickness (cIMT), a surrogate marker of atherosclerosis (13,14). This combination of atherogenic lipid profile and abnormal vascular function may contribute to the known excess atherosclerotic risk of hypothyroidism (15).

Here, reasoning that lipid metabolism in RTHβ might be deranged similarly to hypothyroidism, we have measured circulating lipids and tissue (hepatic, myocellular) lipid content in an unselected cohort of adult patients with this disorder. We also measured other parameters (systemic insulin sensitivity, vascular compliance, endothelial function, and cIMT) to assess atherosclerosis risk in the disorder. We have compared these measurements in RTHβ with the same parameters in age, gender, and body mass index (BMI)-matched healthy control subjects and also compared lipid profiles in patients with RTHβ and TT.

## Methods

### Subjects and study protocols

Adult patients with RTHβ referred to our center over two decades, harboring diverse TRβ mutations, and the age, gender, BMI, and *THRB* genotype of patients, together with measurements undertaken, are listed in Supplementary Table 1 (16). Control metabolic measurements were made in an unselected cohort of healthy adult subjects with normal thyroid function. Thyrotoxic patients comprised individuals with uncontrolled Graves’ disease (as defined by elevated TH with raised antithyroid-stimulating hormone [TSH] receptor antibody levels). In all 3 groups, individuals with diabetes mellitus, history of alcohol excess, known liver disease, or use of lipid-lowering drug therapy treatment were excluded. All measurements were undertaken following an overnight fast. Five RTHβ patients with coexistent autoimmune thyroid disease and 1 with previous partial thyroidectomy were thyroxine treated to maintain normal TSH levels. All investigations were undertaken either as part of ethically approved protocols (RTHβ: Cambridgeshire, LREC 98/154; TT, REC 05/Q0108/117; reference metabolic measurements in healthy subjects, REC 06/Q0108/84), with prior informed consent of patients and participants. Vascular function measurements in healthy subjects were undertaken under the auspices of a separate protocol (REC 05/Q0108/262). Measurements in all groups were undertaken contemporaneously at the time of their recruitment to studies.

### Biochemical measurements

Thyroid hormones (free thyroxine [FT4], free triiodothyronine [FT3], TSH) were measured using a DELFIA® fluoroimmunometric assay (Wallac, Milton Keynes, UK). Lipid profiles were measured by Advia Centaur (Siemens, Germany); nonesterified fatty acids (NEFA), by Roche Free Fatty Acids (Roche Diagnostics, Mannheim, Germany); and insulin by Diasorin XL Liason methods, with calculation of HOMA-IR from fasting plasma glucose and insulin concentrations using the following formula: HOMA-IR = fasting plasma glucose (mmol/L) × fasting plasma insulin (mU/L)/22.5, as described previously (5). Plasma proprotein convertase subtilisin/kexin type 9 (PCSK9) levels were measured using an enzyme-linked immunosorbent assay (R&D Systems, Abingdon, UK). Apolipoprotein B (ApoB) levels were measured using an immunoturbidimetric assay (Sentinel Diagnostics, Milano, Italy).

### Magnetic resonance spectroscopy

Magnetic resonance spectroscopic measures of intrahepatic lipids (IHL) and intramyocellular lipid (IMCL) were obtained using a whole-body Siemens 3T scanner. For IHL, a ^1^H spectrum was measured in a 1.5-cm voxel from the posterior aspect of the right lobe of liver, avoiding blood vessels and biliary tree, using the point resolved selective spectroscopy sequence as described previously (17). IMCL measurements were obtained of the soleus muscle using the point resolved selective spectroscopy sequence with a repetition time of 5 s, an echo time of 35 ms, and 64 averages. Spectra were analyzed as described previously (18), and IMCL was quantified relative to the creatine at 3.0 ppm.

### Vascular function

Studies of vascular function were conducted with subjects supine and at rest in a temperature-controlled room. Endothelial function was assessed by measuring flow-mediated vasodilatation (FMD) of the brachial artery in response to an endothelium-dependent stimulus (reactive hyperemia in response to 5 min of arterial occlusion) as described previously (19). Ultrasound measurements of right and left common carotid intima-media thickness (10 mm longitudinal section, 1 cm from bifurcation) were undertaken as previously described (20). Pulse wave velocity (PWV) was derived by sequentially recording electrocardiogram-gated carotid and femoral waveforms to calculate aortic PWV (aPWV) using a high-fidelity micromanometer embedded within a pulse waveform analysis device (SphygmoCor), with data being corrected for mean arterial pressure as described previously (21).

### Statistical analysis

GraphPad Prism 8.0.1 and Microsoft Excel were used to analyze data. Parametric data are presented as mean +/− SEM, and nonparametric data as median and range. Comparisons of data were undertaken using Mann-Whitney U tests for nonparametric data (triglyceride levels, HOMA-IR values) or 2-tailed Student *t* tests for parametric data (all other measurements; paired for thyrotoxic groups pre- and post-treatment, otherwise nonpaired). Comparisons between groups were performed using 1-way analysis of variance analyses. *P* values of less than 0.05 were considered significant. Correlation coefficients (r^2^) were generated using the Pearson correlation test. For vascular data, aPWV was adjusted for mean arterial pressure and augmentation index for height and heart rate, by univariate analysis of variance.

## Results

### Lipid profiles, ApoB, and PCSK9 levels

Fasting lipid profiles, together with circulating ApoB and PCSK9 levels, were measured in as many RTHβ patients as possible referred to our center over 2 decades. These measurements were compared with parameters in healthy subjects and patients with TT, matched for age, gender, and BMI (Table 1).

**Table 1.** Age, gender, BMI, and thyroid hormone profiles of control, RTHβ, and thyrotoxic patients

	Control	RTHβ	Thyrotoxic	ANOVA	*P* value (RTH β vs. Control)	*P* value (RTH β vs. Thyrotoxic)
Number of patients	100	77	40			
Gender	41M, 59F	27M, 50F	11M, 29F	NS	NS	NS
Age, years	39.06 ± 1.11	40.52 ± 1.74	42.38 ± 1.71	NS	NS	NS
BMI, kg/m^2^	26.54 ± 0.39	26.98 ± 0.56	25.9 ± 0.9	NS	NS	NS
TSH, mU/L	1.9 ± 0.13	4.28 ± 0.63	<0.03 ± 0	<0.01	<0.01	<0.01
FT4, pmol/L	13.47 ± 0.19	32.68 ± 1.32	56.44 ± 3.82	<0.01	<0.01	<0.01
FT3, pmol/L	5.17 ± 0.09	12.2 ± 0.50	29.67 ± 2.79	<0.01	<0.01	<0.01

Data Expressed as mean ± SEM. *P* values less than 0.05 were considered significant. Abbreviations: RTH β, Resistance to Thyroid Hormone beta; M, male; F, female; BMI, Body Mass Index; NS, not significant

**Figure 1.**
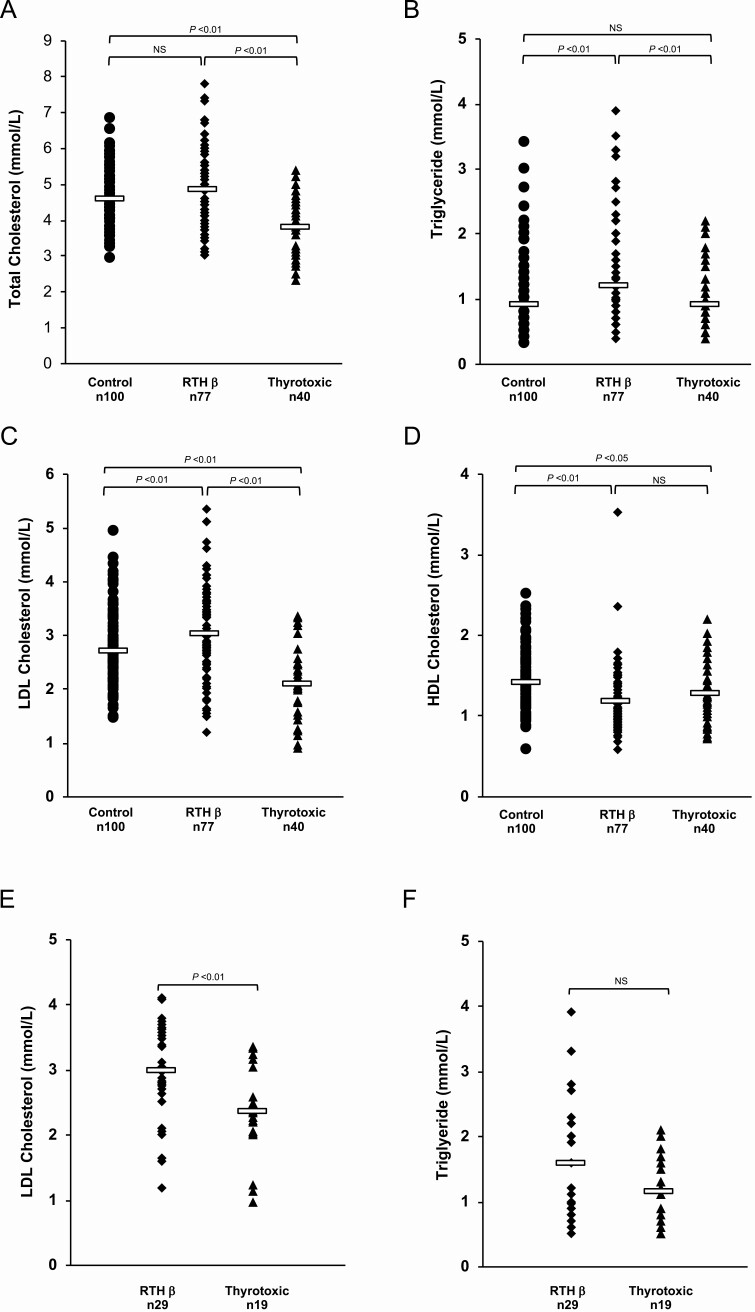
Total cholesterol, triglyceride, LDL and HDL cholesterol levels in healthy controls, RTHβ and thyrotoxic patients (A-D), matched for age, gender, and BMI; LDL and triglyceride levels in RTHβ and thyrotoxic patients, additionally matched for FT4 and FT3 levels (E, F). Each data point represents one patient’s measurement, with bars indicating mean (A, C, D, E) or median (B, F) of the group. NS, not significant.

In RTHβ patients, circulating total cholesterol (Fig. 1A), triglyceride (Fig. 1B) and LDL-C (Fig. 1C) levels were higher, and higher-density lipoprotein cholesterol (HDL-C) concentrations were lower (Fig. 1D) in comparison to control subjects. In contrast, compared to controls, thyrotoxic patients exhibited markedly lower total (Fig. 1A) and LDL-C (Fig. 1C) levels, with comparable triglyceride (Fig. 1B) and slightly lower HDL-C (Fig. 1D) levels. When additionally matched for elevated TH levels (RTHβ mean FT4 39 pmol/L, TT mean FT4 42 pmol/L, *P* = 0.4; RTHβ mean FT3 15.7 pmol/L, TT mean FT3 18.3 pmol/L, *P* = 0.1), circulating levels of LDL-C remained significantly elevated (Fig. 1E) in RTHβ (2.97 mmol/L) than in TT (2.34 mmol/L, *P* < 0.01), and although triglyceride levels remained elevated, this was not significant (Fig. 1F, *P* = 0.2). Clinically significant mixed dyslipidemia (total cholesterol >5mmol/L and/or triglyceride >1.7mmol/L) was present 49% (38/77) of RTHβ patients. Mean circulating LDL-C levels exhibited a tendency to correlate inversely with *THRB* genotype, being more elevated in RTHβ patients with severely deleterious type 1 TRβ mutations (n = 25; R320H, R320C, R338W, R438H, R438C, P453T, P453S, r = −0.73, *P* = 0.06). There was no correlation between triglyceride concentrations and *THRB* genotype for type 1 mutations (r = −0.12, *P* = 0.8). Since patients with only 3 different type 2 mutations (R243W, R383C, R383H) were available, it was not possible to meaningfully assess correlations with triglyceride or LDL-C levels; see Supplementary Figure 1A and 1B (16).

**Figure 2.**
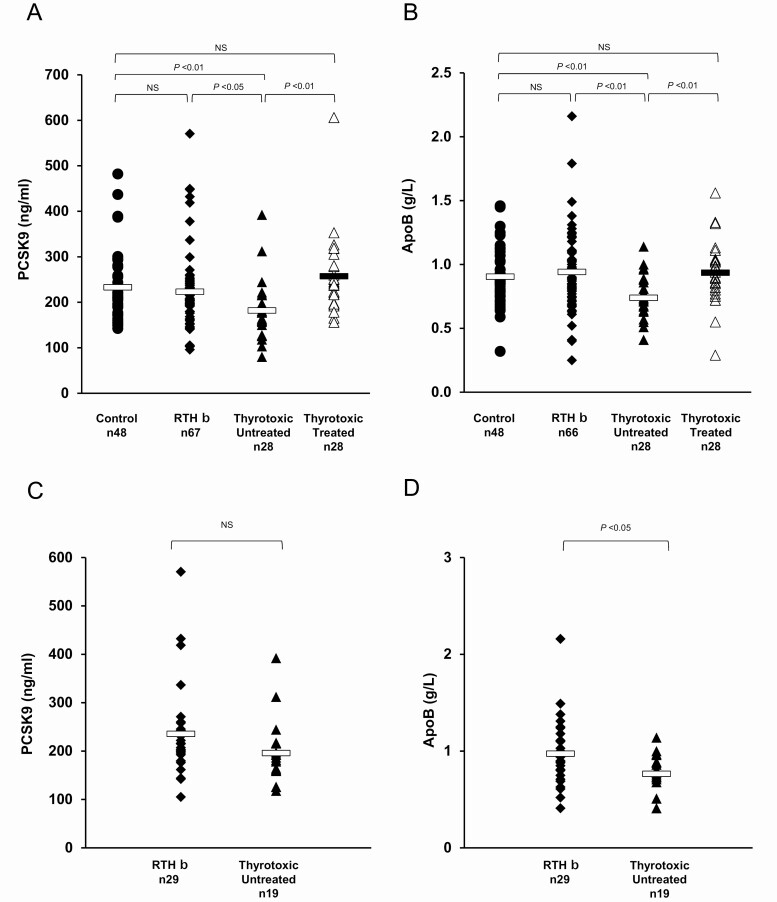
PCSK9 and ApoB levels in healthy controls, RTHβ and thyrotoxic patients, before and after treatment (A, B). Subjects were matched for age, gender, and BMI. PCSK9 and ApoB levels in RTHβ and thyrotoxic patients, additionally matched for FT4 and FT3 levels (C, D). Each data point represents one patient’s measurement, with bars indicating mean of the group. NS, not significant.

Levels of PCSK9 were markedly reduced in TT (TT mean 181 ng/mL *vs* control 232 ng/mL, *P* = 0.002) and rose significantly following treatment (TT mean posttreatment 257 ng/mL, *P* = 0.0001) (Fig. 2A). In contrast, PCSK9 levels in RTHβ patients were not different to control subjects (control mean 232 ng/mL *vs* RTHβ mean 223 ng/mL, *P* = 0.533) and were significantly higher than in thyrotoxic patients (TT mean 182 ng/mL *vs* RTHβ mean 223 ng/mL, *P* = 0.002) (Fig. 2A).

We measured ApoB, recording significantly reduced circulating levels in TT (TT mean 0.74 g/L *vs* control mean 0.9 g/L, *P* = 0.00096) which and normalized (mean 0.94 g/L) following treatment (Fig. 2B). In contrast, ApoB levels in RTHβ patients were not different to control subjects (control mean 0.9 6g/L *vs* RTHβ mean 0.94 g/L, *P* = 0.46), but were significantly higher than in thyrotoxic patients (*P* = 0.0013) (Fig. 2B). When matched for elevated TH levels, both PCSK9 (Fig. 2C, *P* = 0.12), and ApoB levels were higher in RTHβ than TT, but only ApoB levels (Fig. 2D) were significantly different (RTHβ group 0.97 g/L; TT group 0.76 g/L, *P* < 0.05).

### Intrahepatic and intramyocellular lipid, circulating free fatty acids, and systemic insulin sensitivity

Measurements of IHL and IMCL, whole body insulin sensitivity (HOMA-IR), and circulating NEFA were undertaken in subgroups of RTHβ patients (IHL and IMCL n = 28; HOMA-IR n = 59; NEFA n = 66) and compared with parameters in healthy control subjects matched for age (*P* = 0.09), gender (*P* = 0.17), and BMI (*P* = 0.07). Magnetic resonance spectroscopy measurements from subjects deemed to be of reduced reliability due to presence of prominent extramyocellular lipid (for IMCL, 16 of 28 RTHβ subjects) or exceptionally high lipid content (IHL, 1 of 28 RTHβ subjects) were excluded.

**Figure 3.**
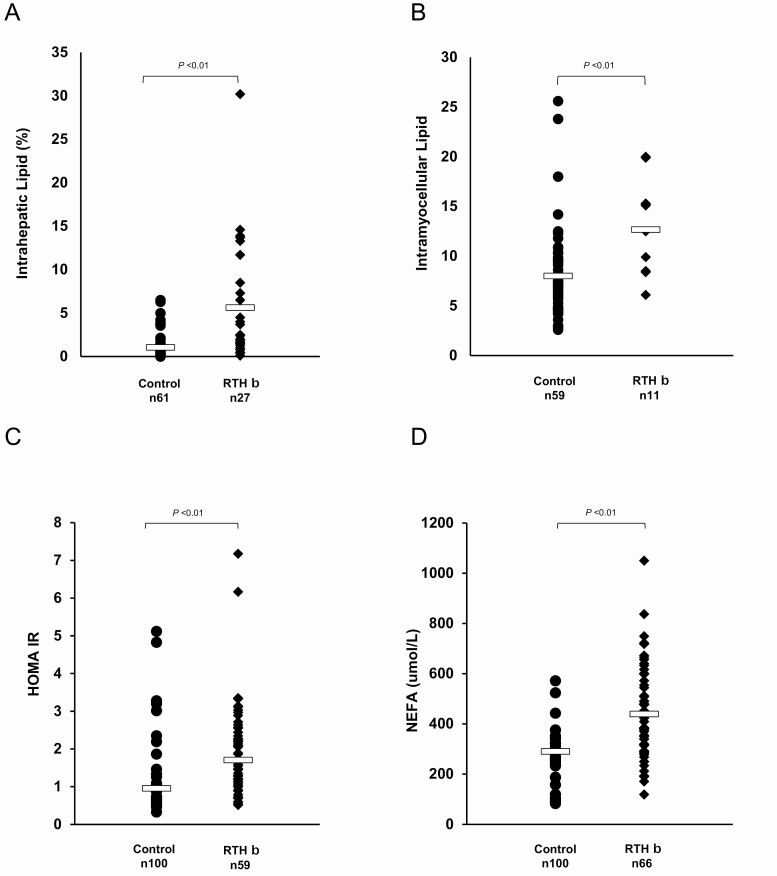
Intrahepatic lipid content (A), intramyocellular lipid content of the soleus muscle (B), HOMA-IR values (C) and NEFA levels (D) in healthy controls and RTHβ patients. Subjects were matched for age, gender, and BMI. Each data point represents one patient’s measurement, with bars indicating mean (A, B, D) or median (C) of the group.

In comparison to healthy controls, both IHL (RTHβ 5.6% *vs* control 1.1%, *P* = 0.02 × 10^–4^, Fig. 3A) and IMCL (RTHβ 12.7 *vs* control 8.0, *P* = 0.002, Fig. 3B) content was significantly higher in RTHβ patients. HOMA-IR, an index of whole-body insulin sensitivity in which higher values denote insulin resistance, was significantly greater in RTHβ (median RTHβ 1.706 *vs* control 0.95, *P* = 0.01 × 10^–2^, Fig. 3C) and this difference persisted even when 2 patients with particularly high HOMA-IR values (7.17, 6.16) were excluded from analysis (median HOMA-IR RTHβ 1.67, *P* = 0.01 × 10^–2^). Circulating NEFA concentrations were significantly raised in RTHβ patients (mean 428 umol/L) *vs* Control (mean 290 umol/L, *P* = 0.01 × 10^–2^) (Fig. 3D).

Fasting plasma glucose (measured in 71 RTHβ patients) was impaired (5.6-6.9 mmol/L, mean 5.8 mmol/L) in 6 RTHβ patients and in the diabetic range (8.5 mmol/L) in one patient. Hemoglobin A1c (measured in 34 RTHβ patients) was abnormal (43 mmol/mol, n = 2; 45 mmol/mol, n = 1) in 3 patients. Overall, glycemic status was abnormal in 12.6% (9 out of 71) of RTHβ patients in whom this was assessed.

### Vascular function

**Figure 4.**
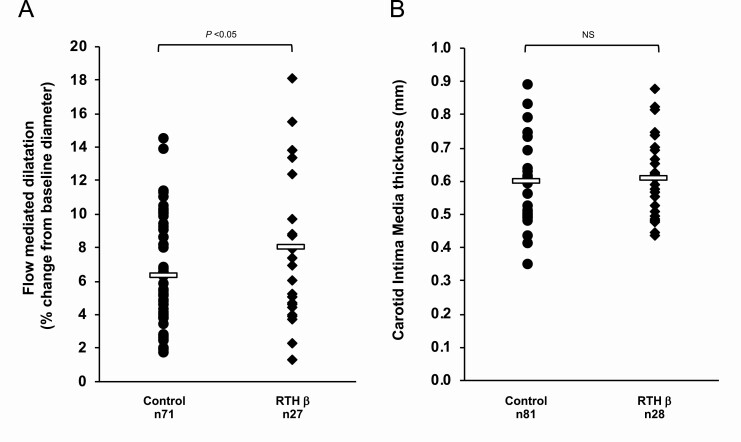
Flow mediated dilatation of the brachial artery (A) and carotid intima media thickness (B) in healthy controls and RTHβ patients. Subjects were matched for age, gender, and BMI. Each data point represents one patient’s measurement, with bars indicating mean of the group. NS, not significant.

Following arterial occlusion, the change in brachial artery caliber or FMD, an endothelium-dependent vascular response, was greater in RTHβ patients than control subjects (RTHβ mean FMD 7.97%, *vs* control mean FMD 6.29%, *P* = 0.04, Fig. 4A). Measurements of cIMT did not differ between either RTHβ patient and control groups overall (RTHβ mean 0.6mm *vs* control mean 0.61 mm, *P* = 0.71, Fig. 4B), or in a subset (RTHβ n = 12, mean 0.66 mm; control n = 15, mean 0.66 mm; *P* = 0.8) of older subjects (age >40 yrs) from these groups.

Surrogate markers of central arterial stiffness and wave reflections, comprising measurements of aPWV (RTHβ aPWV mean 6.746 m/sec *vs* control mean aPWV 6.833 m/sec, *P* = 0.81; see Supplementary Figure 2A, (16) and AIx (RTHβ mean 20.1% *vs* control mean 20.3%, *P* = 0.95; see Supplementary Figure 2B, (16) were not significantly different between RTHβ patients and healthy control subjects.

## Discussion

We have shown that, in comparison to healthy control subjects, patients with RTHβ exhibit raised circulating cholesterol (total and LDL-C) and triglycerides, reduced HDL-C levels and systemic insulin sensitivity, together with increased hepatic lipid and IMCL. Interestingly, this mixed dyslipidemia and ectopic lipid deposition, analogous to features seen in metabolic syndrome, is associated with increased endothelium-dependent vascular reactivity but markers of arterial compliance (PWV, AIx) and atherosclerosis (carotid IMT) are unaltered in the disorder.

Our finding of raised circulating cholesterol concentrations in RTHβ, coupled with previous knowledge that its levels fall less readily following T3 administration in patients (22), support the notion of hepatic resistance to TH action and a relatively hypothyroid status of this target organ in the disorder. In accordance with a previous study, we have found that circulating levels of PCSK9, an enzyme that prevents recycling of the LDL-C receptor to the cell surface, are markedly reduced in TT (23), but remain unaltered in RTHβ patients. This finding suggests that unchanged PCSK9 levels in RTHβ patients, associated with reduced cell surface low-density lipoprotein (LDL) receptor expression and circulating LDL-C clearance, might contribute to the raised LDL-C levels seen in this disorder.

Hyperthyroidism is known to reduce circulating levels of ApoB, a liver-derived apolipoprotein that reflects circulating atherogenic lipoproteins (very low density lipoprotein, intermediate-density lipoprotein, small dense LDL) (10,23) and administration of eprotirome, a liver-selective thyroid hormone receptor agonist, lowers circulating ApoB and triglyceride levels (24). In contrast to low ApoB levels in thyrotoxic patients, we have documented unaltered ApoB levels in RTHβ patients, suggesting that failure to lower ApoB synthesis due to hepatic resistance may mediate observed predilection to raised circulating triglyceride levels in RTHβ. We have shown that circulating free fatty acid levels are elevated in RTHβ, perhaps reflecting increased lipolysis in TRα-expressing adipose tissue. This observation, coupled with knowledge that hypothyroidism reduces hepatic oxidation of free fatty acids (25), raises the possibility that diversion of this fatty acid substrate to lipogenesis may contribute to increased triglyceride synthesis in RTHβ.

Mean IHL content (5.6%) of our RTHβ patient cohort was significantly higher than in age-, gender-, and BMI-matched control subjects (1.1%). Fatty liver correlates inversely with lower T4 levels in the population (26) and is associated with overt and subclinical hypothyroidism (11); intriguingly, in the liver of nonalcoholic fatty liver disease patients, the most highly dysregulated genes are those whose expression is controlled by TH (27). Excess hepatic lipid accumulation, increased lipogenic enzyme gene expression, and diminished fatty acid oxidation has been documented in transgenic mice harboring a dominant negative TRβ mutation (TRβ-PV) known to cause RTHβ (28). Together, these observations suggest that that the risk of hepatic steatosis may be increased in RTHβ and support the notion of hepatic resistance and relative hypothyroid status of liver contributing to its pathogenesis.

Our observations of reduced insulin sensitivity (HOMA-IR) and raised IMCL in RTHβ extend and confirm our previous findings in a smaller cohort of patients (5). Potential mechanisms for raised IMCL include elevation of circulating free fatty acids in RTHβ overwhelming the oxidative capacity of skeletal muscle or raised thyroid hormones acting on this TRα-expressing tissue to upregulate a known target gene (eg, malic enzyme), to promote myocellular lipogenesis (29). Raised IMCL is often associated with systemic insulin resistance and it is conceivable that they may be causally linked; specifically, accumulation of IMCL may be a surrogate marker for excess myocellular deposition of other lipid species (eg, ceramide, diacylglycerol), which are known to perturb insulin action in skeletal muscle (30). Finally, insulin resistance in other contexts (eg, obesity) is frequently associated with hepatic steatosis (31) and could be an additional mechanism contributing to increased IHL in RTHβ.

Higher AIx, a surrogate marker of central artery stiffness and wave reflections, was recorded in a previous small study (n = 12) of RTHβ patients *vs* controls (32), but we found no difference in this parameter in a larger (n = 27) patient cohort when compared to controls. To extend this observation, we went further, assessing central arterial compliance more directly by measuring aPWV and found no significant difference in this larger RTHβ cohort. We have recorded greater FMD of the brachial artery, an endothelium-dependent vascular response, in RTHβ patients. Forearm blood flow is also increased in conventional hyperthyroidism, with such vasodilatation being largely mediated by excessive endothelial nitric oxide production (33). TRα1 is the predominant receptor subtype expressed in human vascular endothelial cells and mediates T3-induced endothelial nitric oxide synthase production (34). Accordingly, we speculate that increased vascular reactivity observed in RTHβ patients might represent endothelial hyperresponsiveness to elevated circulating TH. As cIMT correlates with TSH and FT4 levels within the general population (35) and treatment of hypothyroidism reverses both hypercholesterolemia and increased cIMT in hypothyroidism (14), we were surprised that dyslipidemia in our RTHβ patient cohort was not associated with altered cIMT. However, as our study was cross-sectional, rather than longitudinal, and undertaken in a relatively young (mean age 40 years) RTHβ patient population, we cannot discount the possibility of their atherogenic lipid profile exerting a deleterious vascular effect later in life.

Overall, our observations indicate that monitoring of lipid profiles and hepatic lipid content in RTHβ, possibly focusing particularly on individuals with highly deleterious mutant TRβ genotypes, is warranted, instituting lipid-lowering therapy in patients with hypercholesterolemia and recommending lifestyle changes (such as increased physical activity, weight loss) in individuals with features (eg, central adiposity, hypertriglyceridemia and low HDL-C, insulin resistance) associated with metabolic syndrome. In the future, resmetirom (MGL-3196), a thyroid hormone receptor β selective agonist that has been shown to not only reduce hepatic fat but also lower circulating LDL-C and triglyceride levels in adult NASH patients (36), might represent rational therapy in RTHβ, targeted at patients with mixed dyslipidemia and increased hepatic lipid. How the atherogenic lipid profile, together with enhanced vascular endothelial function and normal cIMT documented here, combines with absence of hypercoagulability we have recorded previously (8) to determine overall cardiometabolic risk in RTHβ remains to be defined.

## Data Availability

Some or all of the data generated or analyzed during this study are included in this published article or in the data repositories listed in the references.

## Additional Information

***Disclosure Summary:*** The authors have no competing financial or other conflicts of interest to declare.
